# Vitreomacular interface after anti-VEGF injections in diabetic macular edema

**DOI:** 10.1186/s40942-021-00295-8

**Published:** 2021-03-19

**Authors:** Carlos E. Veloso, Daniel N. Brocchi, Rishi P. Singh, Márcio B. Nehemy

**Affiliations:** 1 Department of Ophthalmology, Federal University of Minas Gerais, Avenida Nossa Senhora do Carmo 90, Savassi, Belo Horizonte, MG 30330-000 Brazil; 2 Center for Ophthalmic Bioinformatics, Cole Eye Institute, Cleveland Clinic, Cleveland, OH USA

**Keywords:** Diabetic retinopathy, Vascular endothelial growth factor, Vitreomacular adhesion

## Abstract

**Background:**

The purpose of this study was to evaluate the incidence of vitreomacular adhesion (VMA) release after anti-VEGF therapy for the treatment of diabetic macular edema (DME) and to evaluate further changes in outcome.

**Methods:**

This was a retrospective study that enrolled 66 eyes of 66 patients with DME who presented with VMA diagnosed by spectral-domain optical coherence tomography (OCT) at baseline. VMA was classified as focal (attachment: ≤ 1500 μm) or broad (attachment: > 1500 μm). All patients received at least three monthly intravitreal injections of an anti-VEGF agent. Follow-up visits were performed 1 month after each injection to evaluate the incidence of VMA release.

**Results:**

The mean patient age was 61.4 years (range: 29 to 78 years), and 72.7 % were male. The mean best-corrected visual acuity was 0.62 logMAR, and the mean central retinal thickness (CRT) was 473 μm at baseline. The mean length of follow-up was 18.5 months, and the mean number of injections was 5.8. The intravitreal drugs used were aflibercept (40.9 %), ranibizumab (37.9 %) and bevacizumab (21.2 %). Forty-seven eyes had broad VMA, and 19 had focal VMA. Twenty-two eyes (33.3 %) developed VMA release following a mean of 5.7 injections (range: 3–13). Sixteen eyes (72.7 %) with focal VMA and 6 eyes (27.3 %) with broad VMA at baseline developed VMA release. Twenty-one eyes that developed VMA release showed an improvement in CRT following VMA release (mean: -106 μm; range: 22 to 289 μm).

**Conclusions:**

VMA release occurs in approximately 1/3 of patients with DME following anti-VEGF therapy. Most of them show a short-term decrease in CRT.

## Background

Diabetic macular edema (DME) is the most common cause of visual impairment in patients with diabetes mellitus, occurring in approximately 7–8 % of the diabetic population [1]. The pathogenesis of this condition is complex and multifactorial. Previous studies have shown high levels of vascular endothelial growth factor (VEGF) in the retina and vitreous of eyes with DME, suggesting that VEGF may play a role in the development of the disease [2]. Furthermore, several recent randomized clinical trials have shown better visual outcomes with anti-VEGF agents than with classic focal/grid laser treatment for DME 3–5]. In fact, most of the patients involved in these trials presented a gain in visual acuity (VA) of 10 or more letters. A recent study suggested that baseline factors associated with better visual outcomes include lower hemoglobin A1c levels, younger age, and absence of prior panretinal photocoagulation [6].

Many studies have evaluated the influence of vitreomacular adhesion (VMA) on the response to anti-VEGF agents in macular diseases. A recent retrospective study did not identify an association between vitreoretinal interface status and treatment outcomes with anti-VEGF agents for macular edema secondary to retinal vein occlusions [7]. In cases of neovascular age-related macular degeneration (AMD), most of the studies point to a worse VA outcome or the need for more intravitreal injections in eyes with VMA [8–10]. Recently, the effect of VMA on anti-VEGF treatment in cases of DME was evaluated, and it was shown that, unlike neovascular AMD, DME patients with VMA have a greater potential for improvement in visual outcomes with anti-VEGF therapy [11].

Since the configuration of the vitreomacular interface in macular diseases seems to have an effect on visual outcomes, it would be important to know the incidence of VMA release following intravitreal injections of anti-VEGF agents, which could be an independent factor on its own. A recent study showed that only 5.6 % of neovascular AMD patients developed posterior vitreous detachment (PVD) following intravitreal anti-VEGF therapy [12]. It is not established whether intravitreal injection of anti-VEGF agents can induce VMA release in cases of DME. Therefore, the purpose of this study was to evaluate the incidence of VMA release induced by intravitreal injections of anti-VEGF agents and its eventual effect on central retinal thickness (CRT) after this event in cases of DME.

## Methods

This was a retrospective study designed to evaluate the incidence of PVD after intravitreal injections of currently used anti-VEGF drugs for DME. All subjects were informed about the nature of the study and signed a written informed consent in accordance with the tenets of The Declaration of Helsinki.The Ethics Committee of the Federal University of Minas Gerais in Belo Horizonte, Brazil, approved the study.

### Patients

From March 2011 to June 2019, all patients newly diagnosed with DME at the Institute of Vision were enrolled in this study. At baseline, all patients underwent a complete ophthalmological examination, including slit lamp biomicroscopy, color fundus photography, fluorescein angiography and optical coherence tomography (OCT). Inclusion criteria were (a) presence of intraretinal cysts and/or subretinal fluid detected by SD-OCT located at least 750 μm from the central fovea or central retinal thickness ≥ 300 μm; (b) presence of VMA; (c) visual acuity ≥ 20/800 at baseline; (d) follow-up of at least 3 months; and (e) random enrollment of only one eye into this study. If both eyes were eligible, the study eye was randomly selected for entry.

The exclusion criteria were (a) eyes with other conditions known to affect the vitreomacular interface, such as retinal vascular disease, age-related macular degeneration, uveitis and pathologic myopia; (b) ocular conditions that may affect visual acuity; (c) vitreomacular traction; (d) eyes previously submitted to pars plana vitrectomy (PPV) and (e) eyes previously treated with laser, anti-VEGF or corticosteroids for the last three months.

### Vitreomacular interface analysis

**Fig. 1:**
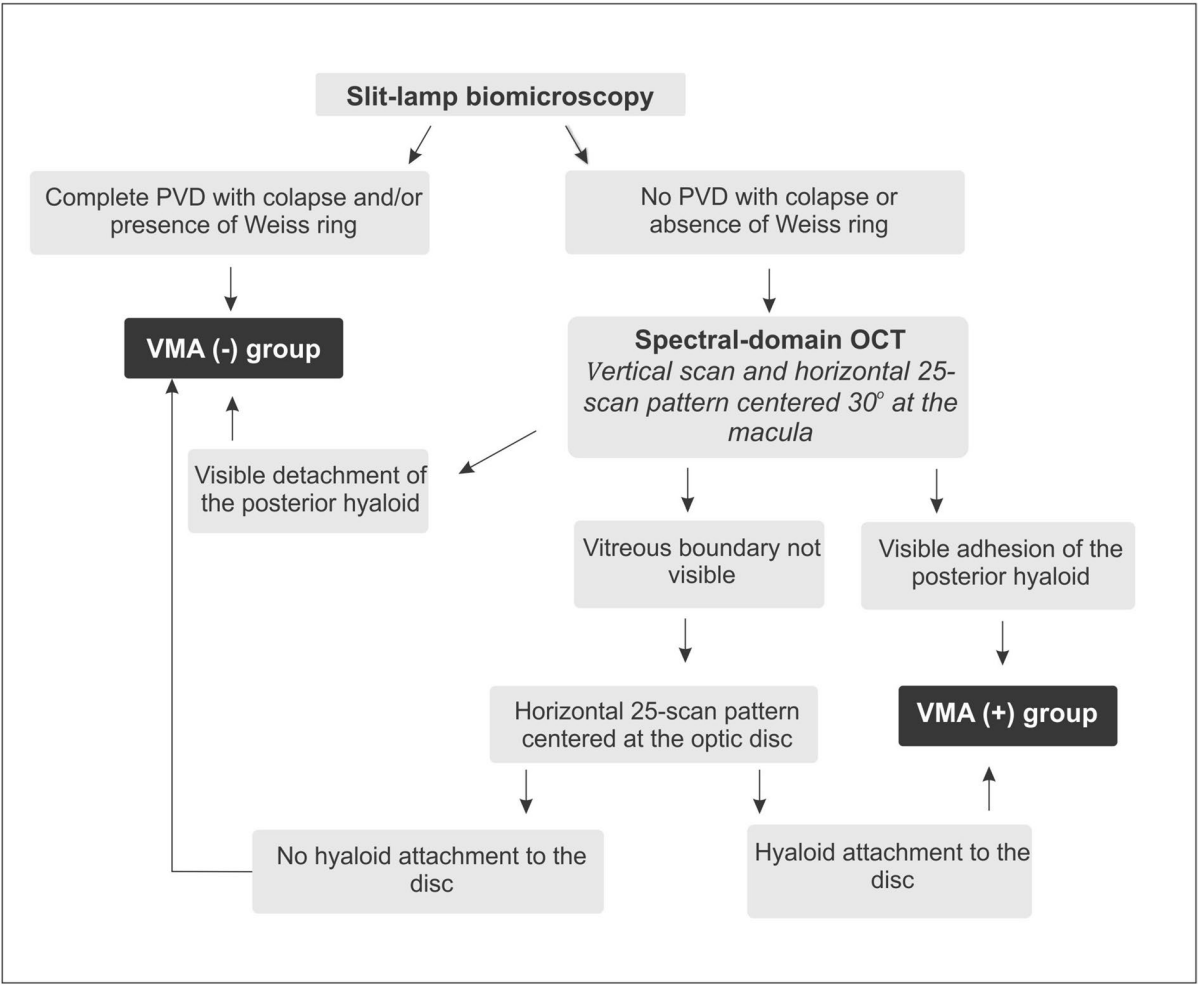
Flowchart used to evaluate vitreomacular adhesion

The evaluation of the vitreomacular interface was based on the flowchart shown in Fig. 1. First, the vitreous condition was analyzed by slit-lamp biomicroscopy. If a complete PVD with collapse was noted and/or a Weiss ring was observed, the eye was considered to present PVD and was included in the VMA(-) group. If none of these characteristics was found, the vitreomacular classification was based on spectral-domain OCT (Spectralis OCT™ [Heidelberg Engineering, Heidelberg, Germany]) analysis. The VMA(+) group included patients who presented visible adhesion of the posterior hyaloid involving the scanned area. For this evaluation, a vertical scan and a horizontal 25-scan pattern centered 30° at the macula were performed. If a detached posterior hyaloid was observed over the macula, the eye was included in the VMA(-) group. However, if the vitreous boundary was not visible, a horizontal 25-scan pattern centered at the optic disc was performed to determine whether the vitreous was attached. If any attachment to the disc was found, the eye was included in the VMA(+) group. In contrast, if no hyperreflective line was attached to the disc and/or macula, vitreous detachment was presumed, and the eye was included in the VMA(-) group. Based on The International Vitreomacular Traction Study Group Classification, patients with VMA were classified according to the diameter of vitreous attachment to the macular surface, with attachment of 1500 μm or less defined as focal and attachment of more than 1500 μm defined as broad [13]. Different certified OCT technicians performed the exam, and two retinal specialists (C.E.V., D.N.B.) analyzed the results. Each retinal physician was masked to the classification determined by the other. If there was any disagreement, they evaluated the data simultaneously with a third investigator (M.B.N.) and came to a consensus. OCT was performed at baseline and at 1 month after each anti-VEGF treatment.

### Anti‐vascular endothelial growth‐factor treatment

Intravitreal anti-VEGF injections were performed using bevacizumab (1.25 mg/0.05 ml; Avastin, Genetech Inc.), ranibizumab (0.5 mg/0.05 ml; Lucentis, Genentech Inc.) or aflibercept (2 mg/0.05 mL; Eylea, Bayer). All intravitreal anti-VEGF injections were performed in the operating room with aseptic techniques, including the prophylactic use of topical iodopovidone 5 %. All patients were subjected to a treatment protocol that included a loading dose with three intravitreal injections of anti-VEGF agents at one-month intervals. After the third dose, they followed a *pro re nata* regimen. Furthermore, after the first injection, there was the possibility of switching of the intravitreal drug in some cases. Retreatment criteria were (a) persistence of or increase in intra- or subretinal fluid and (b) worsening of at least one line of VA. A single retinal specialist (M.B.N.) performed all intravitreal injections.

### Visual acuity and central retinal thickness measurement

Best-corrected visual acuity (BCVA) and CRT were measured before and 1 month after each intravitreal injection. BCVA was determined using a Snellen chart and then converted to logarithm of the minimal angle of resolution (logMAR) values. CRT was measured based on central 1 mm subfield thickness. The automated segmentation of retinal boundaries was used and, in case of any segmentation error, corrected manually.

## Results

A total of 280 eyes of 196 patients were diagnosed with DME during the study period. Of these eyes, 128 eyes (45.7 %) had VMA, and a total of 66 eyes of 66 patients met the inclusion criteria and were evaluated in this study. The mean age was 61.4 years (range: 29 to 78 years), and 47 out of 66 patients (72.7 %) were male. The mean baseline BCVA was 0.62 logMAR, and the mean baseline CRT was 473 μm. Fifty-three eyes (80.3 %) were phakic, and 13 (19.7 %) were pseudophakic. Forty-three eyes (65.1 %) were treated with focal laser treatment before or during the course of the study. The mean follow-up period was 18.5 months (range: 3 to 42 months). The mean number of intravitreal injections was 5.8 (range: 3 to 21 injections). The intravitreal drugs used in the study were aflibercept (40.9 %), ranibizumab (37.9 %) and bevacizumab (21.2 %). Of 66 eyes with DME and VMA, 47 (71.2 %) had broad VMA, and 19 (28.8 %) had focal VMA at baseline.

**Fig. 2:**
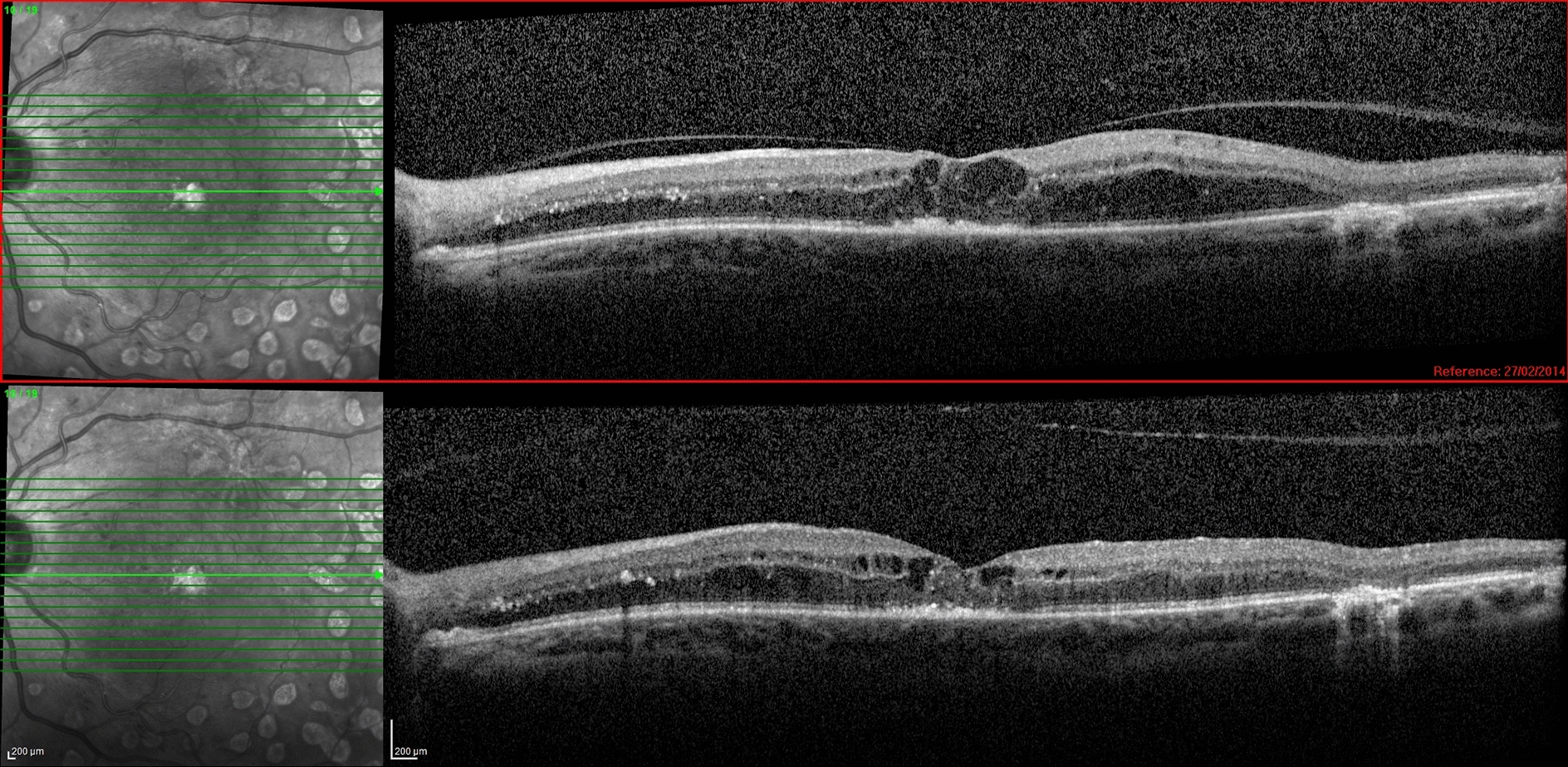
Vitreomacular adhesion release following intravitreal injection of an anti-VEGF agent

A total of 22 eyes (33.3 %) presented VMA release following a mean of 5.7 intravitreal anti-VEGF injections (range: 3–13), while 44 eyes (66.7 %) maintained persistent VMA. VMA release developed after the intravitreal injection of aflibercept in 11 eyes, ranibizumab in 6 eyes and bevacizumab in 5 eyes. CRT decreased at the following visit in 21 eyes that developed VMA release after intravitreal injection (mean CRT reduction: 106 μm; range: 22 to 289 μm) (Fig. 2). Seven out of 22 eyes that developed VMA release after anti-VEGF injection showed improvement in BCVA at the following visit (mean BCVA improvement: 0.2 logMAR; range: 0.1–0.4 logMAR). In the remaining 15 eyes, BCVA did not show any change at the following visit after VMA release, although they had a decrease in CRT. Subsequent evaluations after VMA release showed that some eyes maintained anatomical and/or functional improvement, while others showed a decrease in VA and/or an increase in CRT, during the long-term follow-up.

Patients who developed VMA release in the study eye had a mean age of 61.2 years. Seventeen eyes (77.3 %) that presented VMA release were phakic, and 5 (22.7 %) were pseudophakic. Among 22 eyes that developed VMA release, 16 eyes (72.7 %) had focal VMA at baseline, with diameters of vitreous attachment ranging from 365 to 1120 μm (mean: 618 μm); 6 eyes (27.3 %) were classified as having broad VMA at baseline. The area of attachment in all eyes classified as having broad VMA at baseline showed a progressive reduction prior to the development of VMA release, so that all eyes presented focal VMA by the time this release occurred.

## Discussion

The role of the vitreomacular interface in the pathogenesis and outcome of DME has been previously studied but is not completely understood. Previous studies indicate that DME may be exacerbated due to persistent vitreomacular traction, a thickened and taut posterior hyaloid that may or may not be adherent to the internal limiting membrane, macular traction due to tractional proliferative membranes, or loculation of cytokines in the premacular vitreous pocket [14].

There are two landmark papers dealing with DME and the vitreous in clinical terms. Hikichi et al. showed that if PVD occurs in the presence of DME, it leads to resolution of edema and improvement in visual acuity. In their report, spontaneous resolution of DME was seen in 55 % of eyes in which PVD developed compared with only 25 % in those with persistent VMA [15]. Gaucher et al. showed that the prevalence of VMA was significantly higher in eyes with DME, suggesting that adhesion of the vitreous to the fovea is a risk factor for this disease [16].

PPV has been proposed for the treatment of DME in select cases. After PPV, VEGF and other proinflammatory cytokines would be expected to diffuse away from the macula more easily. Therefore, PPV can produce structural and functional improvements in some eyes with DME. However, a recent meta-analysis showed that the visual gains are not significantly better than those obtained with laser or observation [17].

More recently, several studies evaluated the effects of VMA on anti-VEGF treatment for neovascular AMD, suggesting that VMA may be associated with a worse VA outcome or the need for more intravitreal injections [8–10]. It would be reasonable to suppose that this same relationship might be found in eyes with other macular diseases, including diabetic retinopathy. Therefore, VMA release possibly induced by intravitreal injections of drugs commonly used to treat DME could favor the outcome.

In the present study, 45.7 % of the eyes presented VMA, a higher percentage than those in other recent studies of DME that reported a prevalence ranging from 20.9 to 37.5 % [11, 12, 14–18]. This could be partially explained by different criteria for the diagnosis of VMA. The higher prevalence of VMA in our study may be due to the inclusion of patients with totally attached posterior hyaloid. Other studies considered VMA only when a smaller area of perifoveal vitreoretinal attachment was present, i.e., partial PVD [11, 18]. In addition, previous studies indicate that female sex, previous cataract surgery, advanced age and laser photocoagulation are risk factors for PVD [19–22]. In our study, the majority of patients were male (72.7 %), were phakic (80.3 %), had a mean age of 61.4 years (range: 29 to 78 years) and were treated with focal laser treatment before or during the course of the study (65.1 %).

We found that among 66 eyes with VMA, a total of 22 eyes (33.3 %) developed VMA release following intravitreal anti-VEGF injections, while 44 eyes (66.7 %) maintained persistent VMA. Since all eyes developed VMA release within a relatively short period after intravitreal injection, this indicates that rather than being coincidental, the release in these cases might have been induced by the intravitreal injection. However, it is not clear whether the VMA release occurred based on a pharmacologic or a mechanical effect of the injection. In fact, many intravitreal procedures, including gas injections, can lead to VMA release. For a better analysis of this subject, it would be ideal to have a control group receiving sham injections, which, obviously, would be unethical. The incidence of VMA release after treatment was higher in our study than in previous investigations. A recent study based on data from the READ-3 (Ranibizumab for Edema of the Macula in Diabetes: Protocol 3 with High Doses) clinical trial retrospectively evaluated the role of VMA in visual and anatomic outcomes in patients with DME treated with 6 monthly injections of ranibizumab. They noted that at month 6, among the 26 VMA(+) eyes, 7 eyes (26.9 %) developed PVD [11]. Another published manuscript evaluated patients with DME treated with four monthly ranibizumab injections, with an additional two injections following this, if necessary. At 12 months of follow-up, VMA release occurred in approximately 25 % of that cohort [18]. It is possible that the higher incidence of VMA release in our study may be partially explained by the longer follow-up for most of our patients (mean: 18.5 months). The percentage of VMA release following intravitreal drug injection in eyes with DME was much higher than that in eyes with neovascular AMD (32.4 % vs. 5.6 %). This suggests a stronger VMA in eyes with neovascular AMD [12]. It is possible that chronic low-grade inflammation, scarring or the consequences of chronic exudation in AMD cause the vitreous to become more adherent in the macular region [23]. Nevertheless, the reason for this difference is not clear at present.

Previous studies showed that the majority of patients with either diabetic retinopathy or neovascular AMD presented with a broad area of vitreomacular attachment when they were selected for anti-VEGF therapy. Regarding diabetic patients, a previous study showed that among 26 eyes with DME and VMA at baseline, 5 eyes (19.2 %) had focal VMA, and 21 eyes (80.8 %) had broad VMA [11]. Regarding neovascular AMD, it was recently demonstrated that among 125 eyes with VMA, 10 eyes (8.0 %) were classified as having focal VMA, and 115 eyes (92.0 %) showed broad VMA at baseline [12].

In our study, of 28 eyes that developed VMA release, 16 eyes (72.7 %) had broad VMA, and 6 eyes (27.3 %) had focal VMA at baseline. However, the area of attachment in all eyes classified as having broad VMA showed a progressive reduction prior to the development of its release, so that all eyes presented focal VMA by the time VMA release occurred. In a previous study, 4 of the 5 eyes (80.0 %) that had focal VMA at baseline developed PVD, whereas 3 of the 21 eyes (14.3 %) that had broad VMA at baseline developed PVD (p = 0.01) [11]. In another study involving patients with neovascular AMD, all 7 eyes that developed PVD were classified as having focal VMA at baseline [12]. As expected, these findings suggest that focal attachments are more prone to develop VMA release in both AMD and diabetic retinopathy. The release of VMA is a progressive event, and anti-VEGF agents may, at most, accelerate it. Therefore, as observed in this study, anti-VEGF agents do not have an acute effect on VMA weakening. In contrast, our study showed that eyes with broad VMA showed progressive weakening over months until the VMA became focal and prone to be released.

In our study, CRT decreased in almost all eyes that developed VMA release after intravitreal injection, suggesting that this release may favor short-term anatomical outcomes. Even though this could have a benefit in a short-term period, if left untreated, macular edema may recur, which highlights the complex and multifactorial pathophysiology of this disease. We believe that a short-term evaluation immediately after VMA release, as performed in this study, could offer a more reliable analysis of the influence of this factor on the therapeutic response. In terms of functional improvement, BCVA improved in only 1/3 of the eyes that presented VMA release. It is known that other prognostic factors could influence the functional outcome, including lower hemoglobin A1c levels, younger age and absence of prior panretinal photocoagulation [6]. Although the analyses of these findings could be useful, the present study was not designed with this purpose, and therefore, these data could not be conveniently evaluated.

Considering that, in our study, anti-VEGF agents could be switched after the first injection, it was not possible to know the effect of each drug alone. For this purpose, it would be ideal to analyze subgroups of patients, each one treated with only one type of anti-VEGF agent. Furthermore, the data in this study do not allow us to know whether VMA release after intravitreal anti-VEGF injection would be influenced by the stage of diabetic retinopathy. Studies involving a larger number of patients and a longer follow-up would be necessary for such an assessment. Future investigations are necessary to better establish the possible functional and anatomical long-term benefit of VMA release in eyes with DME treated with anti-VEGF agents.

## Conclusions

In conclusion, we showed that approximately one-third of eyes with DME treated with anti-VEGF agents presented VMA release during the study period. VMA release leads to short-term anatomical improvement.

## Data Availability

The datasets used and/or analysed during the current study are available from the corresponding author on reasonable request.

## Abbreviations

AMD: age-related macular degeneration.
CRT: central retinal thickness.
DME: diabetic macular edema.
OCT: optical coherence tomography.
PPV: pars plana vitrectomy.
PVD: posterior vitreous detachment.
VA: visual acuity.
VEGF: vascular endothelial growth factor.
VMA: vitreomacular adhesion.
